# Nicotinamide Inhibits Aphid Fecundity and Impacts Survival

**DOI:** 10.1038/s41598-019-55931-z

**Published:** 2019-12-23

**Authors:** Sampurna Sattar, Mario T. Martinez, Andres F. Ruiz, Wendy Hanna-Rose, Gary A. Thompson

**Affiliations:** 1 College of Agricultural Sciences, The Pennsylvania State University, University Park, PA 16802 United States of America; 2 Department of Biological Sciences, Alcorn State University, Lorman, MS 39096 United States of America; 3 Department of Biochemistry and Molecular Biology, The Pennsylvania State University, University Park, PA 16802 United States of America

**Keywords:** Biotic, Entomology

## Abstract

Nicotinamide (NAM) alters behavior in *C. elegans* and Drosophila, serving as an agonist of TRPV channels affecting sensory neurons and mimicking the mode of action of insecticides used to control phloem-feeding insects. The impact of NAM on green peach aphid (*Myzus persicae*) behaviors was assessed in artificial diet assays and foliar applications to Arabidopsis plants. Aphids feeding on artificial diets supplemented with NAM impaired stylet movement causing feeding interruptions and ultimately starvation and death. Aphid feeding behaviors were negatively impacted on NAM sprayed plants at concentrations as low as 2.5 mM leading to increased mortality. In choice assays with NAM sprayed leaves aphids showed clear preference for untreated control leaves. NAM is an intermediate in the NAD salvage pathway that should accumulate in nicotinamidase (*nic*) mutants. LC-MS analysis showed NAM accumulates 60-fold in *nic-1-1* Arabidopsis mutants as compared with Col-0. Aphid reproductive potential was significantly decreased on *nic-1-1* mutant plants, resulting in a smaller colony size and arrested population development. The results support the hypothesis that dietary NAM causes behavioral changes in aphids, including altered feeding, reduced reproduction, and increased mortality. NAM is thought to bind to TRPV channels causing overstimulation of sensory neurons in the aphid feeding apparatus.

## Introduction

Nicotinamide (NAM) is ubiquitous metabolite found in all living organisms as an intermediary compound in the synthesis of the coenzyme nicotinamide adenine dinucleotide (NAD). In plants, free NAM in the cell is converted to nicotinic acid (NA) that is either cycled back to the NAD salvage pathway or metabolized into methyl derivative of NA (MeNA), converted to plant hormone Trigonelline (Tg), NA N-glucoside and/or NA O-glucoside^1^. Among these, MeNA and NA O-glucoside are both precursors of NAD, with MeNA taking part in long distance transport and NA O-glucoside being the NAD precursor in storage tissues^2^. Most commonly NAD functions as a redox carrier essential for growth and development. In addition, NAD functions as a signaling molecule during plant stress, inducing defenses in response to plant pathogens^3,4^. In the presence of oxidative stress NAD is readily depleted by consuming enzymes such as PARP and sirtuins to produce NAM^5,6^. Elevated levels of NAM act as a feedback inhibitor of the DNA-strand breaking enzyme PARP and histone deacetylase sirtuins, stabilizing NAD levels in the stressed tissue^6^. Excess NAM is either shunted into the salvage biosynthetic pathway (Preiss-Handler pathway) to produce NAD or processed into NA-derivatives. NAD biosynthesis and its salvage pathway are conserved across species, and NAM is cycled back through the NAD salvage pathway in plants, animals, bacteria, and fungi^1^.

NAM, its derivative NA, and their synthetic analogs isonicotinamide and 2,6-dichloro-isonicotinic acid have been implicated in defense responses against biotic stressors^6–8^. Exogenous application of NAM to spruce seeds has been shown to reduce the incidence of pine weevil damage^9^. NAM-induced behavioral responses were observed in *Caenorhabditis elegans* and *Drosophila* exhibiting exaggerated nose bending and diminished response to sound, respectively^10^. It was further shown that these behavioral changes were due to NAM binding TRPV (vanilloid-type transient receptor potential) ion channels in both organisms. In animals, TRPV channels are evolutionarily conserved and regulate a wide array of functions^11^. In Drosophila, these channels have important roles in chemosensation and mechanosensation. Feeding behavior in insects often involves complex interactions of both chemosensory and somatosensory responses, both of which are regulated by TRPV channels^11^. Drosophila TRPV subunit homolog OSM-9 have been shown to detect chemical stimuli in the diet during social feeding in worms^12^ and recently *C. elegans* OSM-9 subunits have been shown to bind NAM^10^.

Pymetrozine, pyrifluquinazon, and afidopyropen are small synthetic molecules commonly used as insecticides. These compounds specifically bind TRPV receptors, impacting sensory functions in sap-sucking insects such as aphids, whiteflies, sharpshooters, psyllids and plant hoppers^13,14^. Electropenetration graph (EPG) studies of aphids exposed to pymetrozine suggested that pymetrozine specifically impacted neurons controlling feeding behavior, causing starvation and ultimately death^15^. Further studies with locusts (*Locusta migratoria*) established pymetrozine as a neuroactive compound that selectively impacts chordotonal mechanoreceptors^16^. Recently, TRPV channels from pea aphid have been shown to bind pymetrozine and afidopyropen^17^. Insect TRPV channels are made up of two heteromeric proteins Nanchung (Nan) and Inactive (Iav), that are unique to the insect chordotonal stretch receptor neurons^13^. Drosophila Nan is more broadly expressed in chordotonal and multidendritic neurons, whereas, Iav is restricted to the chordotonal neurons only. Both proteins bind pymetrozine and afidopyropen in a competitive manner, with the latter showing higher affinity for Drosophila TRPV channels^17^. Although both proteins bind the insecticides, the activity of afidopyropen requires binding to Nan and is enhanced in presence of both Iav and Nan^17^. In contrast, pymetrozine, pyrifluquinazon, activity requires binding to both Nan and Iav. NAM is similar in that its activity also requires binding to both Nan and Iav^10,13^.

In this study, NAM was investigated for its effects on phloem-feeding aphids and its ability to induce behavioral changes that are often regulated by TRPV channels. Since NAM and pymetrozine bind to the same receptor, it was hypothesized that they would have similar behavioral responses in aphids, leading to feeding cessation and resulting in starvation and death.

## Results

### Aphids are negatively impacted by NAM in artificial diet assays

**Figure 1:**
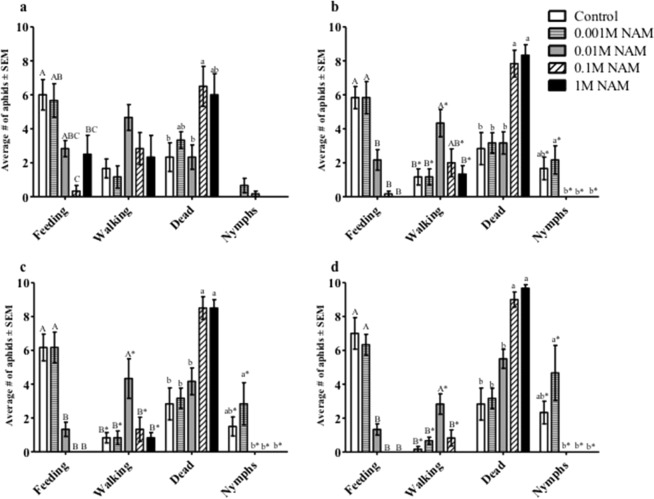
*Myzus persicae* responses to feeding on artificial diets supplemented with ten-fold increases in NAM concentration from 0 to 1 M NAM. Aphid feeding behavior, survival, and fecundity in response to NAM were observed at: (**a**) 12 hours, (**b**) 18 hours, (**c**) 24 hours, and (**d**) 48 hours. The average number of aphids in each behavioral response group were analyzed by one-way ANOVA. Means for each behavioral response that do not share a letter are significantly different at the 95% confidence level.

To determine the effects of NAM in the diet of phloem feeding insects, two polyphagous aphid species, *M. persicae* and *A. gossypii*, were exposed to ten-fold increasing concentrations (0.001 M to 1 M) of NAM in sachet artificial diets and observed for 48-hour time periods (Fig. 1 and Supplementary Fig. S1). After 12 hours, the majority of *M. persicae* aphids were settled and feeding on the control (no NAM) and 0.001 M NAM diets, although some exhibited walking behavior (Fig. 1a). Significantly fewer aphids were observed feeding on diets containing 0.01 M, 0.1 M, and 1 M NAM, corresponding to an increase in mortality observed at the two highest concentrations of NAM. Adult aphids feeding on the control diet and diets containing the two lowest concentrations of NAM (0.001 M, 0.01 M) also began producing nymphs at 12 hours. Nymphs continued to accumulate in the control and 0.001 M NAM diets throughout the 48-hour observation period. Aphid mortality further increased after 18 hours in aphids exposed to 0.1 M or 1 M NAM diet, more than all other treatments including the control. Significantly fewer aphids were feeding on diets containing the three highest NAM concentrations (0.01 M, 0.1 M, 1 M), whereas no difference was observed between the control and 0.001 M NAM diets. Reproduction was also most strongly impacted at the three highest concentrations of NAM. Similar trends in feeding behaviors, mortality, and reproduction were observed after 24 and 48 hours. The minimum effective concentration of NAM appears to be between 0.001 M (similar to 0 M control) and 0.01 M NAM. Similar results were obtained with *A. gossypii* (Supplementary Fig. S1).

**Figure 2:**
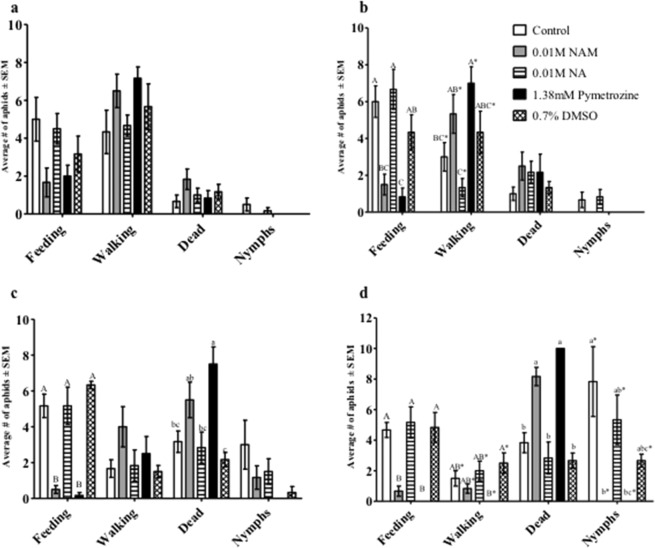
Artificial diet assays with *Myzus persicae* to establish the specificity of aphid responses to NAM. Aphid feeding behavior, survival, and fecundity were observed at: (**a**) 6 hours, (**b**) 12 hours, (**c**) 24 hours, and (**d**) 48 hours in response to feeding on artificial diets without supplementation (Control) or supplemented with 10 mM NAM, 10 mM nicotinic acid (NA), 1.38 mM pymetrozine insecticide solubilized in 0.7% DMSO, or 0.7% DMSO alone. The average number of aphids in each behavioral response group were analyzed by one-way ANOVA. Means for each behavioral response that do not share a letter are significantly different at the 95% confidence level.

The specificity of the minimum effective dose (0.01 M) of NAM was confirmed in an experiment that compared *M. persicae* feeding behaviors, mortality, and reproduction with an equal concentration of nicotinic acid (NA) and 1.38 mM pymetrozine, an aphidicidal compound that affects feeding behaviors by binding to TRPV channels in the insect (Fig. 2). Pymetrozine was solubilized in 0.7% DMSO, therefore another control diet containing 0.7% DMSO was used to account for its effect on the aphids. At six hours (Fig. 2a), a trend of reduced feeding and increased walking was observed for aphids exposed to the NAM and pymetrozine diets; however, the differences among all the treatments were not statistically different. In contrast, at 12 and 24 hours (Fig. 2b,c) significantly more aphids were feeding on control diets as well as diets containing 0.01 M NA and DMSO as compared to diets containing NAM or pymetrozine. Also, higher aphid mortality was observed for NAM and pymetrozine at the same time points as compared to buffer controls and NA diets, in which most aphids were feeding in these diet arenas. By 48 hours (Fig. 2d), 100% and 90% mortality were observed for aphids exposed to diets containing pymetrozine and NAM, respectively. The number of nymphs produced were also negatively affected by these treatments, with pymetrozine and NAM having significantly fewer nymphs than the buffer controls and NA diet.

**Figure 3:**
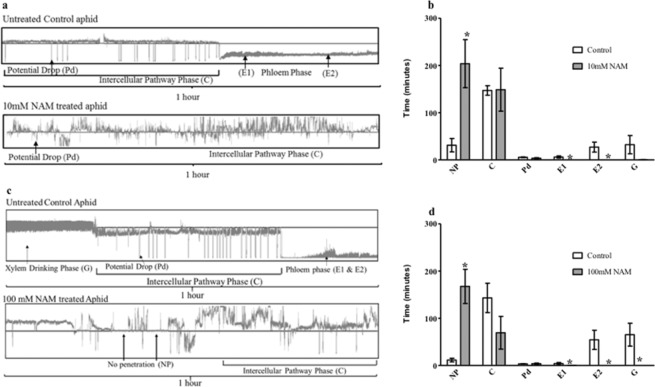
Effects of NAM on *Myzus persicae* feeding behaviors monitored by EPG. Aphids were allowed to feed on artificial diets containing no NAM (Control), 10 mM NAM or 100 mM NAM for 12 hours and then transferred to Arabidopsis Col-0 plants for comparative EPG profiling. EPG parameters included time spent in no penetration (np), intercellular pathway (C), cell puncture (pd), phloem salivation (E1), phloem sap ingestion (E2), and xylem drinking (G). (**a**) Representative EPG waveforms (1-hour duration) of untreated control and 10 mM NAM treated aphids. (**b**) The mean duration in each EPG event using 10 mM NAM was compared to no NAM control. (**c**) Representative EPG waveforms (1-hour duration) of untreated control and 100 mM NAM treated aphids. (**d**) Mean duration of each EPG event recorded from aphids treated with 100 mM NAM and compared to untreated control. Data were statistically analyzed using non-parametric Kruskal-Wallis test (*P ≤ 0.05).

To determine if aphids could resume normal feeding after exposure to NAM, *M. persicae* were fed 10 mM and 100 mM NAM supplemented artificial diet for 12 hours then transferred to Col-0 Arabidopsis plants where feeding behaviors were recorded over a four-hour period by electrical penetration graph (EPG) analysis (Fig. 3). The EPG technique identifies and correlates waveforms to specific movement of the aphid stylet within the host tissue, allowing quantification of an individual stylet activity event and its exact duration. Most insects exposed to 10 mM and 100 mM NAM initiated probing behaviors but did not engage in phloem ingestion (E2) or xylem-associated activities (G), with a few insects failing to engage in any probing behavior (PD). In contrast, aphids feeding on artificial diet without NAM exhibited normal stylet functions, engaging in cell penetration (PD), pathway (C), phloem salivation (E1), phloem ingestion (E2), and xylem-associated (G) activities. Interestingly, aphids feeding on the control diet spent more amount of time drinking from the xylem (G) than ingesting phloem sap when transferred to Arabidopsis plants (Fig. 3b, d). Aphids exposed to the control diet most often transitioned from E1 to E2 (phloem ingestion), whereas those aphids exposed to NAM that engaged in E1 failed to reach E2 phase.

### Foliar application of NAM increases aphid mortality and impacts host choice

**Figure 4:**
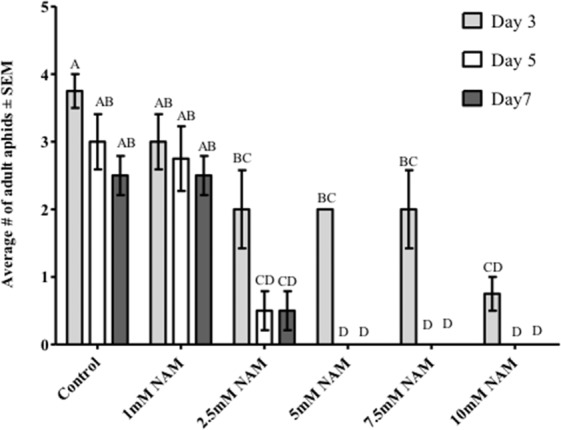
Effect of NAM spray applications to Arabidopsis wild type Col-0 plants. Aqueous solutions of NAM at five concentrations (0, 1.0, 2.5, 5.0, 7.5, 10 mM) were sprayed on five-week old Arabidopsis Col-0 plants and air dried for 12 hours. Four adult *Myzus persicae* placed on cauline leaves were counted after 3 days, 5 days and 7 days after spraying. The average number of aphids in each treatment were analyzed by 2-way ANOVA. Means that do not share a letter are significantly different at the 95% confidence level.

To test whether exogenous foliar applications of NAM impact aphid survival, Arabidopsis plants were sprayed with five concentrations (1 mM, 2.5 mM, 5 mM, 7.5 mM, and 10 mM) of NAM that spanned the effective doses of >1 mM to 10 mM NAM determined in the diet assays. After three days, 50% of the adult *M. persicae* aphids had died on the plants sprayed with 2.5 mM, 5 mM, and 7.5 mM NAM, with 100% aphid mortality occurring after 5 days. Plants sprayed with 10 mM NAM showed 75% mortality at 3 days and 100% mortality at 5 days (Fig. 4). Control plants sprayed with aqueous solution of 0.001% Silwet or plants sprayed with 1 mM NAM did not exhibit significant adverse effects on aphid survival.

**Figure 5:**
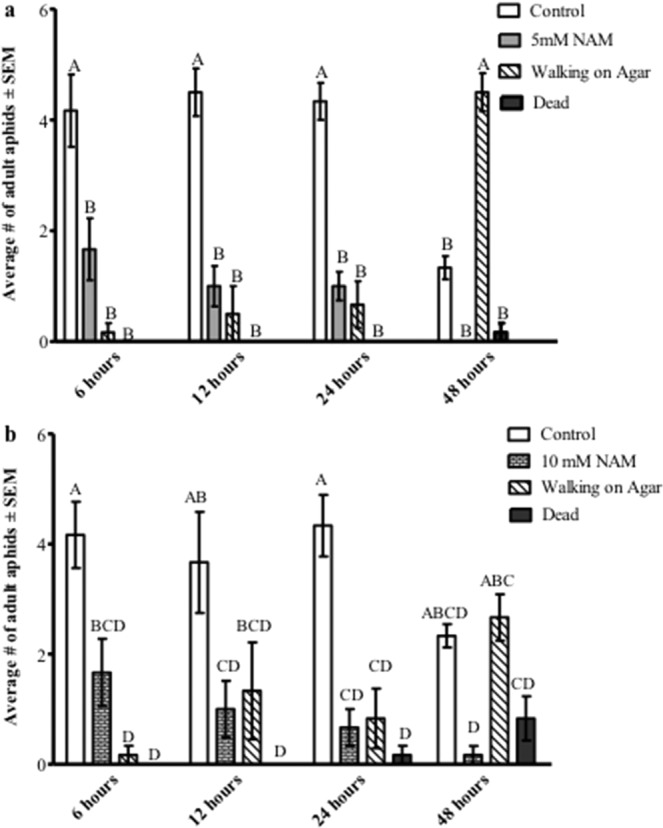
Host preference choice assays. Excised leaves of untreated Arabidopsis Col-0 plants or plants sprayed with (**a**) 5 mM or (**b**) 10 mM NAM were placed on agar in a petri dish and 10 adult *Myzus persicae* were released into the arena. The number of aphids on control leaves, NAM-treated leaves, or walking on agar were counted at 6 hours, 12 hours, 24 hours, and 48 hours after introducing aphids into the arena. The average number of aphids at the different locations were analyzed by 2-way ANOVA. Means that do not share a letter are significantly different at the 95% confidence level.

Choice assays were performed by placing six *M. persicae* aphids in the center of a feeding arena (1% agar plates) containing Arabidopsis leaves excised from plants sprayed with either 5 mM or 10 mM NAM and 0.001% Silwet (control). After a 6-hour period, significantly more aphids were present on the Silwet-sprayed control leaves than the NAM-treated leaves at both concentrations (Fig. 5). Similar observations were recorded after 24 hours; however, after 48 hours more aphids were observed to be walking on the agar than feeding on control leaves and no aphids were present on the 5 mM NAM-treated leaves, suggesting a significant decrease in host quality in all leaves. A similar number of aphids were found on control leaves as walking in the 10 mM NAM arenas at 48 hours with almost no aphids on the 10 mM NAM treated leaves. In all treatments, aphids preferred to be on the untreated control than the NAM treated leaves.

### NAM accumulating in *nic-1-1* Arabidopsis mutants impacts aphid fecundity and feeding behaviors

**Figure 6:**
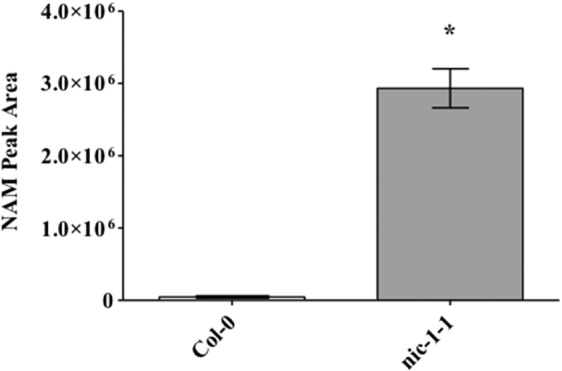
Relative nicotinamide levels measured in the Arabidopsis nicotinamidase mutant *nic-1-1*. Shown are the average LC-MS peak areas indicating relative nicotinamide (NAM) levels plotted for Col-0 and *nic-1-1* mutant. The bars graphs represent average peak area for NAM with standard error of the mean for each genotype. Means that are significantly different at 95% confidence level are denoted by *.

It was previously shown that the Arabidopsis nicotinamidase mutant *nic-1-1* disrupted the NAD salvage pathway preventing the conversion of [^14 ^C]-NAM to NA^18^. Theoretically, this would result in the accumulation of the NAM intermediate in the mutant; however, this had not been experimentally determined. Accumulation of NAM in *nic-1-1* mutant leaf tissue was examined in comparison to wild-type Col-0 by LC-MS. NAM detection by LC-MS was first confirmed by adding a NAM standard to a Col-0 tissue preparation. NAM (123.055 g/mol) was detected in two distinct peaks eluting at 1.02 and 1.4 minutes (Supplementary Fig. S2). The areas under both peaks from unspiked Col-0 and *nic-1-1* samples were combined and then normalized to residual protein/peptide content in the extracted samples for comparative analysis (Fig. 6). Protein content did not differ significantly between the Col-1 and *nic-1-1* extracted samples (average of 288 and 302 μg/ml, respectively). In contrast, results from the LC-MS analysis showed that there was an approximate 60-fold increase in NAM accumulation in the homozygous *nic-1-1* mutant plants (Supplementary Figs. S2 and S3). In previous studies, mutation of the *C. elegans* nicotinamidase resulted in changes to approximately 25% of the annotated metabolome in addition to the 19-fold increase in NAM levels^19^. Similar, likely indirect, changes in the metabolome were observed in *nic-1-1* mutants. A total of seven of the forty-three features (16%) annotated in the LC-MS data changed significantly (P < 0.05) between *nic-1-1* and *Col-0* plants (Supplementary Figs. S2 and S3), but only one (arginine) was of similar magnitude to that observed with NAM.

**Figure 7:**
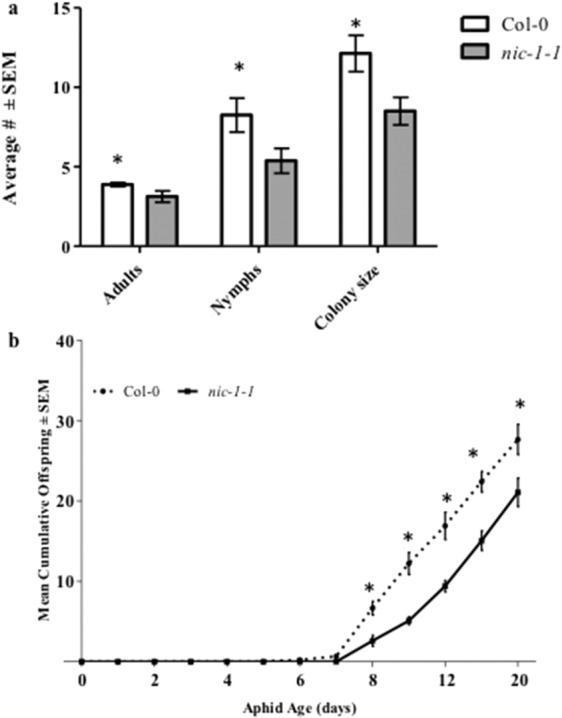
Regulation of colony size of caged *Myzus persicae* aphids on 5-week old homozygous *nic-1-1* mutant and wild type Col-0 plants. (**a**) After 4 days, the number of adults and nymphs were recorded and is represented as the mean (n = 8) with error bars indicating standard error of the mean for each genotype. Data is analyzed by t-test and * indicate significant difference in the mean for each group (P ≤ 0.05). (**b**) Mean pre-reproductive period and fecundity of single *Myzus persicae* aphid reared on homozygous nic-1 mutant and wild-type (Col-0) plants grown at 21 °C under (14 h/10 h light/dark at 40.0 mmol m^−2^ s^−1^ in a randomized block design. The data points represent cumulative mean offsprings at each time point (n = 10) and was analyzed for statistical significance by t-test and * indicate significant difference in the mean for each group (P ≤ 0.05).

To determine if elevated NAM in the *nic-1-1* mutant affected aphid survival and reproduction, four adult *M. persicae* aphids were released on wild type Col-0 and *nic-1-1* mutant plants. The size of the insect colony consisting of adults and nymphs after four days was significantly larger on the Col-0 plants as compared to the *nic-1-1* mutant (Fig. 7a). Adult survival and reproduction were negatively impacted on *nic-1-1*. A detailed analysis of the life cycle of single aphids born on *nic-1-1* mutant or Col-0 plants for a three-week period also revealed the negative effects of NAM on aphid reproduction (Fig. 7). The pre-reproductive period (days from birth to first progeny) was extended an average of 0.7 days, reducing the reproductive period (days producing progeny) by a concomitant 0.67 days in aphids completing their life cycle on the *nic-1-1* mutant plant (Table 1). The total number of aphids born on *nic-1-1* during the three-week period was consistently lower than on wild type Col-0 plants (Fig. 7b). Aphids reared on Col-0 plants had higher daily nymph production (1.372 nymphs/day) compared to *nic-1-1* plants (1.067 nymphs/day). The intrinsic rate of increase (r_m_) of each aphid relates the fecundity of an individual aphid to its development time as an estimate of the population growth on each Arabidopsis genotype. The r_m_ for the aphids on Col-0 (0.345) was significantly different from those on the *nic-1-1* host (0.271), indicating that the colony size on the *nic-1-1* mutant will be smaller compared to Col-0 at a given time.

**Table 1:** Duration of developmental periods and measures of fecundity of *M. persicae* reared on Col-0 or *nic-1-1* plants.

Reproductive parameters	Col-0	*nic-1-1*
Pre-reproductive period (days)	6.5 ± 0.26 a	7.2 ± 0.13 b
Reproductive period (days)	13.55 ± 0.268 a	12.88 ± 0.13 b
Intrinsic rate of increase (r_m_)	0.345 ± 0.025 a	0.271 ± 0.174 b

Means that do not share a letter in each row are significantly different at 5% level.

**Figure 8:**
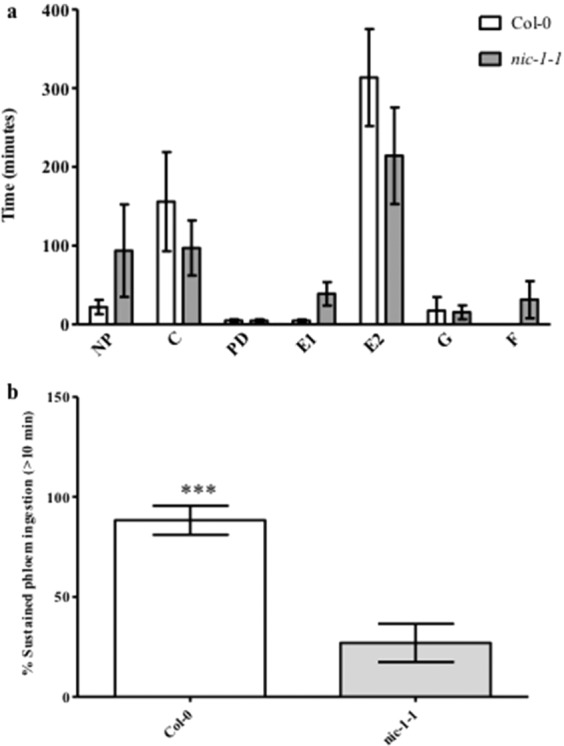
EPG analysis of feeding behaviors of *Myzus persicae* on Arabidopsis Col-0 or *nic-1-1* mutant plants. (**a**) Complete feeding profiles. EPG parameters included time spent in no penetration (np), intercellular pathway (C), cell puncture (pd), phloem salivation (E1), phloem sap ingestion (E2), and xylem drinking (G). (**b**) Comparative time spent in sustained phloem ingestion where individual bouts of phloem sap ingestion (E2) were greater than or equal to 10 minutes in duration (***P < 0.001).

*M. persicae* feeding behaviors on Col-0 and *nic-1-1* plants monitored by EPG analysis for an eight-hour duration also revealed differences in aphid responses to elevated NAM. Overall, aphids feeding on both Col-0 and *nic-1-1* plants appeared to spend similar amount of total time in pathway (C) and phloem ingestion (E2) phases. In contrast, aphids feeding on *nic-1-1* mutant plants spent more time in phloem salivation and pre-sieve element penetration behaviors (E1). Aphids on both genotypes also spent similar amount of time in xylem-associated behaviors (G) and cell punctures (PD) (Fig. 8a). Interestingly, some of the aphids on *nic-1-1* mutant plants also experienced difficulty in stylet penetration (F). While the total time spent on phloem ingestion was not significantly different for the two genotypes, aphids on the *nic-1-1* mutant were primarily feeding on the phloem (E2) for short bursts of less than 10 minutes followed by periods of phloem salivation (E1). Approximately 88% of the E2 phases recorded for aphids on Col-0 plants exceeded 10 minutes indicating sustained phloem ingestion, whereas aphids feeding on *nic-1-1* mutants more often failed to reach sustained phloem ingestion phase with only 22% of the E2 segments lasting more than 10 minutes (Fig. 8b).

## Discussion

A novel role for NAM in modifying behavioral responses has been reported in *C. elegans* and Drosophila^10^. In these organisms, NAM binds to subunits of TRPV ion channels serving as an agonist to modulate sensory responses. Similarly, several commercial insecticides (pymetrozine, pyrifluquinazon, and afidopyropen) also bind to subunits of the TRPV channels. In Drosophila, these insecticides abolish anti-gravitaxis behavior and in aphids, impact their ability to feed by preventing normal functioning of receptor neurons^13,15,17^. Homologs of genes encoding the two TRPV channel subunits Nanchung (Nan) and Inactive (Iav), initially cloned from Drosophila, have been identified in pea aphid^17^ and *M. persicae* genomes (unpublished). Heterologous expression of the pea aphid genes encoding Nan or Iav in CHO-K1 cells have shown that both pymetrozine and afidopyropene bind to and activate TRPV channels^17^.

To initially understand the cross-species impact of NAM-induced behavioral responses, mixed-aged aphids were fed NAM directly in artificial diets and indirectly on plants sprayed with NAM. Altered behavior was observed for two polyphagous aphid species, *M. persicae* and *A. gossypii*, at concentrations above 1 mM NAM added to diets, resulting in reduced aphid feeding and ultimately higher levels of mortality (Fig. 1, Supplementary Fig. S1). In individual experiments both aphid species exhibited similar responses to NAM-supplemented diets; however, between experiments (Figs. 1 and 2) mortality in *M. persicae* varied between 60% to 80% in response to the 10 mM NAM treatment possibly due to the use of mixed-aged aphids that are at different developmental stages. Pymetrozine added to artificial diets induced similar behavioral responses by *M. persicae* as those observed with NAM-supplemented diets (Fig. 2). Spray applications of NAM to Arabidopsis Col-0 plants at dosages as low as 2.5 mM had similar effects to the diet assays, significantly increasing aphid mortality in no-choice assays (Fig. 4) and impacting host preference as observed in choice assays with excised leaves (Fig. 5). Previous reports of pymetrozine-treated host plants showed significantly reduced periods of phloem-sap ingestion for *M. persicae* as well as reduced xylem contact for the xylem feeder glassy-winged sharp shooter (*Homalodisca coagulata*) that resulted in increased levels of insect mortality^15,20^. Similarly, on pymetrozine-treated sweet orange Asian citrus psyllid (*Diaphorina citri*) showed reduced duration in phloem ingestion, decreasing insect survival and acquisition/transmission of *Candidatus* Liberibacter asiaticus^21^.

To monitor the impact of NAM on aphid stylet movement, aphids were fed NAM in artificial diets for 12 hours and then transferred to Arabidopsis Col-0 plants for EPG analysis (Fig. 3). The primary objective of this experiment was to provide a physiologically responsive dose of NAM (10 mM or 100 mM) directly to the aphid and then record aphid feeding behaviors when transferred to a host plant. The experiment was designed to determine if feeding cues presented by the whole plant could overcome the effects of NAM observed on artificial diets, impacting aphid probing as well as passive phloem ingestion. Aphids with no prior exposure to NAM but feeding on artificial diet were able to penetrate the epidermal and parenchyma tissues to reach the phloem. In addition to phloem ingestion, the aphids also spent a significant amount of time ingesting from the xylem, suggesting the aphids experience osmotic stress while feeding on the diet^22^. Prolonged drinking from the xylem was perhaps necessary to adjust the osmotic balance in the aphid tissues and such events were often followed by periods of sustained phloem ingestion. In contrast, aphids feeding on the NAM diet exhibited significantly reduced stylet penetration events and failed to engage in phloem-sap ingestion, phloem salivation, or xylem drinking. Such compromised stylet movement is consistent with pymetrozine-mediated inhibition of stylet penetration and phloem ingestion due to impaired functioning of the cibarial valves, food pump, and the salivary pump^15^. Upadhyay and co-workers^10^ provided evidence for NAM binding to Nan-Iav proteins in the Drosophila TRPV channel, resulting in altered behavioral responses induced by NAM activation of TRPV channels. Although there is no direct experimental evidence for NAM binding to aphid TRPV channels, NAM-induced impaired feeding exhibited by aphids further suggests that mechanosensory coordination of aphid stylet movement is controlled in part by TRPV channels in receptor neurons within aphid gustatory sense organs as proposed for pymetrozine-mediated inhibition of feeding^15,20^.

Impaired stylet function that inhibits feeding could also negatively impact aphid reproduction and delay the growth and development of nymphs into reproductive adults. Aphids feeding on NAM in artificial diets produced fewer nymphs as compared to those feeding on controls (Figs. 1, 2, Supplementary Fig. S1) consistent with reduced fecundity that was previously observed for pymetrozine treatments^15^. Aphids feeding on Arabidopsis *nic-1-1* mutants also showed reduced colony size (Fig. 7a) due to a combination of increased mortality and the production of fewer nymphs. The inability to engage in extended periods of sustained phloem ingestion by aphids in response to elevated NAM in *nic-1-1* mutants (Fig. 8b) is likely responsible for delaying aphid reproductive development and reduced fecundity (Table 1, Fig. 7b). In addition, a slower intrinsic rate of growth (Table 1) is predictive of a significantly smaller colony size on plants that accumulate NAM, which could impact aphid populations in argicultural ecosystems. Interestingly, the impact of NAM on aphids feeding on the *nic-1-1* mutant was much less pronounced than observed in either the artificial diet or spray experiments. This might be due to threshold effects in the effective concentration of NAM *in planta* or the intercellular location of elevated NAM concentrations in leaf tissues. There appears to be a narrow window of the effective concentration between 1 mM (ineffective) and 2.5 mM (effective) of NAM that impacts aphids in exogenous applications.

NAM is an integral component of the salvage pathway that ultimately produces NAD. While NAD functions as a primary acceptor of electrons during metabolic reactions, recent reports provide compelling evidence for extracellular NAD and its derivatives as damage activated molecular patterns (DAMPs) in plants, increasing defense responses against pathogens^3,4^. Enhanced NAD levels directly induce accumulation of salicylic acid (SA) and expression of defense response genes (*PR1*, *chitinases*, and WRKY transcription factors)^23^. Increased expression of *PR1* transcripts and enhanced levels of SA are induced by *M. persicae* infestation in Arabidopsis^24^; however, SA mediated defense responses does not impact *M. persicae* interactions with Arabidopsis^25^. Exogenous applications of NAM to Col-0 leaves could increase NAD concentrations through the salvage pathway, but such an increase would not be expected to result in an effective defense response against aphids. Furthermore, nicotinamidase mutants (*nic-1-1*) disrupt the salvage pathway reducing NAD levels^18^ and increasing NAM concentrations in the leaf tissues. It is possible that NAM itself functions as a DAMP in planta, inducing an effective defense response by a yet to be defined mechanism. However, the rapid behavioral responses to elevated NAM in the diet along with the intriguing commonalities with insecticides that bind TRPV channels in aphids suggest a direct role for NAM inducing aphid mortality and loss of fecundity by directly impacting aphid feeding behaviors.

## Material and Methods

### Plant growth and insect rearing conditions

Chinese cabbage and *Myzus persicae*: Chinese cabbage plants were grown in controlled conditions of 23 °C, 60% relative humidity and a photoperiod of 16∶8 hours (light∶dark) for 3–4 weeks until the leaf length reached 5–7 cm. A clonal colony of *Myzus persicae* (green peach aphid) was maintained on Chinese cabbage plants at 25 ± 1 °C, 50–60% relative humidity, and a photoperiod of 16∶8 hours (light∶dark).

Honeydew melon and *Aphis gossypii*: Honeydew melon plants were grown in controlled conditions of 23 °C, 60% relative humidity and a photoperiod of 16∶8 hours (light∶dark) for four weeks until they reached the four-leaf stage. A clonal colony of *A. gossypii* (cotton melon aphid) was maintained on susceptible honeydew melon plants in a controlled growth chamber at 21 °C, with a photoperiod of 16∶8 hours (light∶dark).

Arabidopsis: *Arabidopsis thaliana* ecotype Columbia (Col-0) and the T-DNA insertion (Col-0 background) mutant *nic-1-1*^18^ were grown for five weeks in controlled growth conditions at 21 °C with a photoperiod of 14:10 hours (light:dark) at 40.0 µmol m^−2^ s^−1^.

### *In vitro* aphid feeding assays

*In vitro* feeding assays were conducted with *M. persicae* or *A. gossypii* in sterilized 25 ml glass beakers (4.5 cm depth × 2.5 cm diameter). An artificial diet sachet was created by applying 75 µl of complete aphid synthetic diet^26^ sandwiched between two parafilm layers, sealing the mouth of the beaker. Ten aphids of mixed developmental stages were placed in the beakers and allowed to feed on the artificial diet for 48 hours. Initial experiments were designed to determine the effective doses over a wide range of nicotinamide (NAM) concentrations. Artificial diets were supplemented with ten-fold increasing concentrations (0 to 1 M) of NAM. To establish the specificity of the *M. persicae* response to NAM, diets were supplemented with 10 mM NAM, 10 mM nicotinic acid (NA, negative control), 1.38 µM pymetrozine solubilized in 0.7% DMSO (Millipore-Sigma, positive control), 0.7% DMSO (solvent control) or with diet alone (buffer control). The number of aphids in and outside the diet arena (feeding vs. walking behaviors), aphid mortality, and the number of nymphs (reproduction) were recorded at regular intervals between 2–48 hours. Each assay consisted of five biological replicates and the differences in the treatment for each behavior were measured using one-way ANOVA followed by Tukey’s test (*P* < 0.05).

### Foliar treatment of NAM

Arabidopsis Col-0 plants were sprayed with an aqueous solution of NAM (with 0.001% Silwet) at concentrations of 0, 1, 2.5, 5, 7.5 and 10 mM until runoff. Control plants were sprayed with aqueous solution of 0.001% Silwet. Plants were allowed to dry for 12 hours. For no-choice assays, four adult *M. persicae* aphids were placed on rosette leaves. The numbers of aphids on the plants were monitored after three, five, and seven days post-spraying. Each treatment group consisted of four biological replicates and the differences in the treatment were statistically analyzed with two-way ANOVA with grouping by Tukey’s test using 95% confidence interval. For choice assays, Col-0 plants were sprayed with aqueous solution (with 0.001% Silwet) without NAM (control) or with 5 mM or 10 mM NAM and allowed to dry as described above. Single excised rosette leaves were placed on 1% agar plates; each agar plate (100 mm × 20 mm) was divided equally into two segments along the diameter and each segment received a NAM-treated leaf (5 mM or 10 mM) or control leaf. Ten adult aphids were placed in the center of the plate between the excised leaves, which was designated as the aphid arena. The number of aphids on the NAM-treated or control leaves or in the arena were monitored for 48 hours. Each group consisted of eight biological replicates. Differences between groups were analyzed by two-way ANOVA followed by Tukey’s test using 95% confidence interval.

### Relative accumulation of NAM in *nic-1-1* mutant

Targeted examination of NAM was performed at the Metabolomics Core Facility at The Pennsylvania State University by a modified version of ion pairing reversed phase positive ion electrospray ionization method using LC-MS^27^. Homogenized leaf tissue (approximately 100 mg) from Col-0 or *nic-1-1* mutant plants (n = 3) were used for NAM estimation. Briefly, the homogenized tissue was sonicated with extraction buffer (99.875% acidified methanol with 0.125% formic acid) in a water bath set at 20 °C for 15 minutes. Following sonication, the samples were centrifuged for 10 minutes at maximum speed and the resulting supernatant was divided into two fractions for processing; one was used for LC-MS analysis using a SCIEX (Framingham, MA) 5600 TripleTOF® with a Shimadzu (Columbia, MD) Prominence UFLC, and the other was analyzed for protein/peptide content as a normalization method. Raw data files were converted for analysis by MS-DIAL for peak extraction using centroid mode^28^. Parameters for the analysis are included in the supplementary file. Features were identified based on ms/ms data from the MSP library in MS-DIAL. Features with peak fill proportions less than 0.4 were excluded. A number of metabolites appeared to be detected in two peaks, similar to NAM; these were only included if the identity of both peaks could be confirmed with ms/ms data. Peak area for metabolites of interest were normalized to protein content of the extracted samples determined by BCA assay (Thermo Fisher Scientific) and the fold change between genotypes was calculated. Data were transformed using Box-Cox transformation and statistically analyzed by one-way ANOVA with Tukey’s significant difference test using Minitab18.

### Aphid life-history traits

Col-0 (n = 10) or *nic-1-1* mutant (n = 10) Arabidopsis plants were placed in a completely randomized design. A single newborn *M. persicae* nymph was confined to a leaf and observed daily for 21 days. When an aphid became reproductive, newborn nymphs were removed each day and the number of nymphs recorded. Differences in the mean cumulative number of nymphs born to replicated single aphids on each genotype were analyzed by one-way ANOVA. Developmental parameters including the intrinsic rate of increase, pre-reproductive period, and reproductive period were compared between aphids on the two Arabidopsis genotypes. The intrinsic rate of increase (r_m_) was calculated using the formula r_m_ = (Ln(Md) × *c*)/D as described by Wyatt and White^29^ where Md is the number of nymphs produced by the adult in D days, D is the number days till the first reproduction after the occurrence of adult molt, and constant *c* = 0.738 is the approximation of the proportion of the total fecundity produced by the female in the first D days of reproduction. Data were analyzed by one-way ANOVA and the means were compared by Tukey’s test (*P* < 0.05).

### No-choice assay

No-choice assays were used to determine the impact of Col-0 and *nic-1-1* mutant on survival and fecundity of adult *M. persicae* aphids. Four reproductive aphids were place on rosette leaves of either Col-0 or *nic-1-1* mutant plants and covered with plastic cage to prevent escape. The number of adult aphids and nymphs on each plant were counted after two, four, six, and eight days. Each group consisted to eight biological replicates. Differences between groups were analyzed by one-way ANOVA followed by Tukey’s test using 95% confidence interval.

### Feeding behavior

*M. persicae* feeding behavior was analyzed using the electrical penetration graph (EPG) technique. Adult wingless aphids were collected and starved for 1 hour prior to start of the EPG recording. A single aphid was connected to the Giga-8 Direct Current EPG (EPG-Systems, Wageningen, The Netherlands) as described by Tjalingi and co-workers^30^. Data recording was initiated one hour after the insect was placed on the plant and continued for four to eight hours. Parameters such as time spent in phloem sap ingestion (E2 phase), phloem salivation (E1 phase), intercellular pathway phase (C), xylem drinking (G), cell puncture (pd), no penetration (np), and stylet derailment (F) were recorded by the Stylet^+^ software for individual aphids and the mean duration in each parameter was statistically analyzed using the nonparametric Kruskal-Wallis test.

Two experimental approaches were used to study aphid feeding behaviors by EPG in response to NAM. To understand the impact of NAM on aphid stylet function, aphids were exposed to control (no NAM), 10 mM NAM and 100 mM NAM in artificial diets (see above for *in vitro* feeding experiment details) for 12 hours and then allowed to feed on leaves of intact Col-0 Arabidopsis plants where EPG recordings were conducted for four hours. Five recordings were taken for each treatment group (control, 10 mM and 100 mM NAM) and the data were analyzed as described above. EPG experiments were also designed to understand the impact of elevated endogenous levels of NAM on aphid feeding behaviors. Eight-hour EPG recordings of single aphids feeding on Col-0 wild type or *nic-1-1* mutant Arabidopsis plants were conducted. Eight recordings were taken for aphids on individual plants of each genotype and the data were analyzed as described above.

## Supplementary information

Nicotinamide Inhibits Aphid Fecundity and Impacts Survival

## Data Availability

The authors declare that the data supporting the findings of this study are available within the article and its Supplementary Information Files or available from the corresponding author upon reasonable request.

## References

[1] Gakière B (2018). NAD + Biosynthesis and Signaling in Plants. Crit Rev Plant Scic..

[2] Wu R (2018). MeNA, Controlled by Reversible Methylation of Nicotinate, Is an NAD Precursor that Undergoes Long-Distance Transport in Arabidopsis. Mol. Plant..

[3] Pétriacq P (2013). NAD: Not just as pawn on the board of plant-pathogen interactions. Plant Signal Behav..

[4] Poltronieri P, Čerekovic N (2018). Roles of Nicotinamide Adenine Dinucleotide (NAD+) in Biological Systems. Challenges.

[5] Hashida SN, Takahashi H, Uchimiya H (2009). The role of NAD biosynthesis in plant development and stress responses. Ann. Bot..

[6] Berglund T (2017). Nicotinamide; antioxidative and DNA hypomethylation effects in plant cells. Plant Physiol. Biochem..

[7] Basson AE, Dubery IA (2007). Identification of a cytochrome P450 cDNA (CYP98A5) from Phaseolus vulgaris, inducible by 3,5-dichlorosalicylic acid and 2,6-dichloro isonicotinic acid. J. Plant Physiol..

[8] Metraux, J. P. *et al*. Induced Systemic Resistance in Cucumber in Response to 2,6-Dichloro-Isonicotinic Acid and Pathogens. In 432–439 (Springer, Dordrecht, 1991).

[9] Berglund T, Lindström A, Aghelpasand H, Stattin E, Ohlsson AB (2016). Protection of spruce seedlings against pine weevil attacks by treatment of seeds or seedlings with nicotinamide, nicotinic acid and jasmonic acid. Forestry.

[10] Upadhyay A (2016). Nicotinamide is an endogenous agonist for a C. elegans TRPV OSM-9 and OCR-4 channel. Nat. Commun..

[11] Venkatachalam, K., Luo, J. & Montell, C. In Mamm. *Transient Recept. Potential Cation Channels*. (eds. Nilius, B. & Flockerzi, V.) 937–962 (Springer International Publishing, 2014).

[12] De Bono M, Tobin DM, Davis MW, Avery L, Bargmann CI (2002). Social feeding in Caenorhabditis elegans is induced by neurons that detect aversive stimuli. Nature.

[13] Nesterov A (2015). TRP Channels in Insect Stretch Receptors as Insecticide Targets. Neuron.

[14] Wang L-X (2019). Pymetrozine activates TRPV channels of brown planthopper Nilaparvata lugens. Pestic. Biochem. Physiol..

[15] Harrewijn P, Kayser H (1997). Pymetrozine, a Fast-Acting and Selective Inhibitor of Aphid Feeding. In - situ Studies with Electronic Monitoring of Feeding Behaviour. Pestic. Sci..

[16] Ausborn J (2005). The insecticide pymetrozine selectively affects chordotonal mechanoreceptors. J. Exp. Biol..

[17] Kandasamy R (2017). Afidopyropen: New and potent modulator of insect transient receptor potential channels. Insect Biochem. Mol. Biol..

[18] Wang G, Pichersky E (2007). Nicotinamidase participates in the salvage pathway of NAD biosynthesis in Arabidopsis. Plant J..

[19] Wang W (2015). Comparative Metabolomic Profiling Reveals That Dysregulated Glycolysis Stemming from Lack of Salvage NAD+ Biosynthesis Impairs Reproductive Development in Caenorhabditis elegans. J. Biol. Chem..

[20] Bextine BR, Harshman D, Johnson MC, Miller TA (2004). Impact of pymetrozine on glassy-winged sharpshooter feeding behavior and rate of Xylella fastidiosa transmission. J. Insect Sci..

[21] Raj Boina D, Youn Y, Folimonova S, Stelinski LL (2011). Effects of pymetrozine, an antifeedant of Hemiptera, on Asian citrus psyllid, Diaphorina citri, feeding behavior, survival and transmission of Candidatus Liberibacter asiaticus. Pest Manag. Sci..

[22] Pompon J, Quiring D, Goyer C, Giordanengo P, Pelletier Y (2011). A phloem-sap feeder mixes phloem and xylem sap to regulate osmotic potential. J. Insect Physiol..

[23] Pétriacq P (2012). Inducible NAD overproduction in Arabidopsis alters metabolic pools and gene expression correlated with increased salicylate content and resistance to Pst-AvrRpm1. Plant J..

[24] Moran PJ, Thompson GA (2001). Molecular responses to aphid feeding in Arabidopsis in relation to plant defense pathways. Plant Physiol..

[25] Louis J, Shah J (2013). *Arabidopsis thaliana*-*Myzus persicae* interaction:shaping the understanding of plant defense against phloem-feeding aphids. Front. Plant Sci..

[26] Mittler TE, Dadd RH (1962). Artificial Feeding and Rearing of the Aphid, Myzus persicae (Sulzer), on a Completely Defined Synthetic Diet. Nature..

[27] De Vos RC (2007). Untargeted large-scale plant metabolomics using liquid chromatography coupled to mass spectrometry. Nat. Protoc..

[28] Tsugawa H (2019). A cheminformatics approach to characterize metabolomes in stable-isotope labeled organisms. Nat. Methods..

[29] Wyatt IJ, White PF (1977). Simple Estimation of Intrinsic Increase Rates for Aphids and Tetranychid Mites. J. Appl. Ecol..

[30] Tjallingii WF (1985). Electrical nature of recorded signals during stylet penetration by aphids. Entomol. Exp. Appl..

