# Supplementary material for: Nicotinamide Inhibits Aphid Fecundity and Impacts Survival
Source: Sci Rep. 2019 Dec 23;9:19709. doi: 10.1038/s41598-019-55931-z (PMC6928209; doi:10.1038/s41598-019-55931-z)
Supplement: Supplementary file 1 — Nicotinamide Inhibits Aphid Fecundity and Impacts Survival [file 41598_2019_55931_MOESM1_ESM.pdf]

Title: Nicotinamide Inhibits Aphid Fecundity and Impacts Survival

Authors: Sampurna Sattar, Mario T. Martinez, Andres F. Ruiz, Wendy Hanna-Rose and Gary A. Thompson

Supplementary Information

Supplementary Data

Supplementary Material and Methods

LC MS peak calling and data collection parameters

Supplementary Figure S1. *Aphis gossypii* responses to feeding on artificial diets supplemented with NAM concentrations (0 to 1 M NAM). Aphid feeding behavior, survival, and fecundity in response NAM were observed at a. 6 hours, b. 12 hours, c. 24 hours, and d. 48 hours. The average number of aphids in each behavioral response group were analyzed by one-way ANOVA. Means for each behavioral response that do not share a letter are significantly different at the 95% confidence level.

Supplemental Figure S2. LC-MS analysis of NAM in Col-0 wild-type and *nic-1-1* mutant Arabidopsis leaves and accumulation of NAM in both genotypes. a. Chromatograph of extracts from leaf samples from four Col-0 wild-type plants. NAM was detected in samples 1, 2, and 3 at very low intensity levels. b. NAM was readily detected in three (5, 6, 7) leaf samples of *nic-1-1* mutant plants at higher intensity levels relative to control Col-0 samples. Retention times for the two NAM peaks were 1.01 and 1.45 minutes for sample 5, 1.03 and 1.44 minutes for sample 6, and 1.03 and 1.46 minutes for sample 7. c. Col-0 sample 4 was spiked with NAM that eluted in two peaks at retention times of 1.02 minutes and 1.40 minutes. d. Fold-change of metabolites from *nic-1-1* mutants that are significantly ($P \leq 0.01$) different from wild type Col-0. Accumulation of all metabolites was normalized to total protein in the respective samples.

Supplemental Figure S3. Complete list of metabolites detected in *nic-1-1* mutant plants using LC-MS analysis.

Supplementary Material and Methods

LC-MS peak calling and data collection parameters

Retention time begin 0

Retention time end 100

Mass range begin 0

Mass range end 500

Centroid parameters

MS1 tolerance 0.01

MS2 tolerance 0.025

Isotope recognition

Maximum charged number 2

Data processing

Number of threads 4

Peak detection parameters

Smoothing method LinearWeightedMovingAverage

Smoothing level 5

Minimum peak width 5

Minimum peak height 2000

Peak spotting parameters

Mass slice width 0.1

Exclusion mass list (mass & tolerance)

Deconvolution parameters

Sigma window value 0.5

MS2Dec amplitude cut off 0

Exclude after precursor True

Keep isotope until 0.5

Keep original precursor isotopes False

Exclude after precursor True

MSP file and MS/MS identification setting

MSP file C:\Users\MBF\Desktop\PCA Programs\MS-DIAL.reference.Library\MSMS-AllPublic_IROATECH-Curated-Pos_20180301.msp

Retention time tolerance 0.5

Accurate mass tolerance (MS1) 0.01

Accurate mass tolerance (MS2) 0.05

Identification score cut off 80

Using retention time for scoring True

Text file and post identification (retention time and accurate mass based) setting

Text file

Retention time tolerance 0.5

Accurate mass tolerance 0.001

Identification score cut off 85

Advanced setting for identification

Relative abundance cut off 0

Top candidate report True

Adduct ion setting

[M+H]+

[M+NH4]+

[M+Na]+

Alignment parameters setting

Reference file F:\Mario\needs converting\Plant Data18\AQPOS18MAY_05-#26.mzML

Retention time tolerance 0.25

MS1 tolerance 0.015

Retention time factor 0.5

MS1 factor 0.5

Peak count filter 0

QC at least filter False

Remove feature based on peak height fold-change False

Sample max / blank average 0

Sample average / blank average 0

Keep identified and annotated metabolites False

Keep removable features and assign the tag for checking False

Replace true zero values with 1/2 of minimum peak height over all samples False

Tracking of isotope labels

Tracking of isotopic labels FALSE

Supplementary Figure S1.

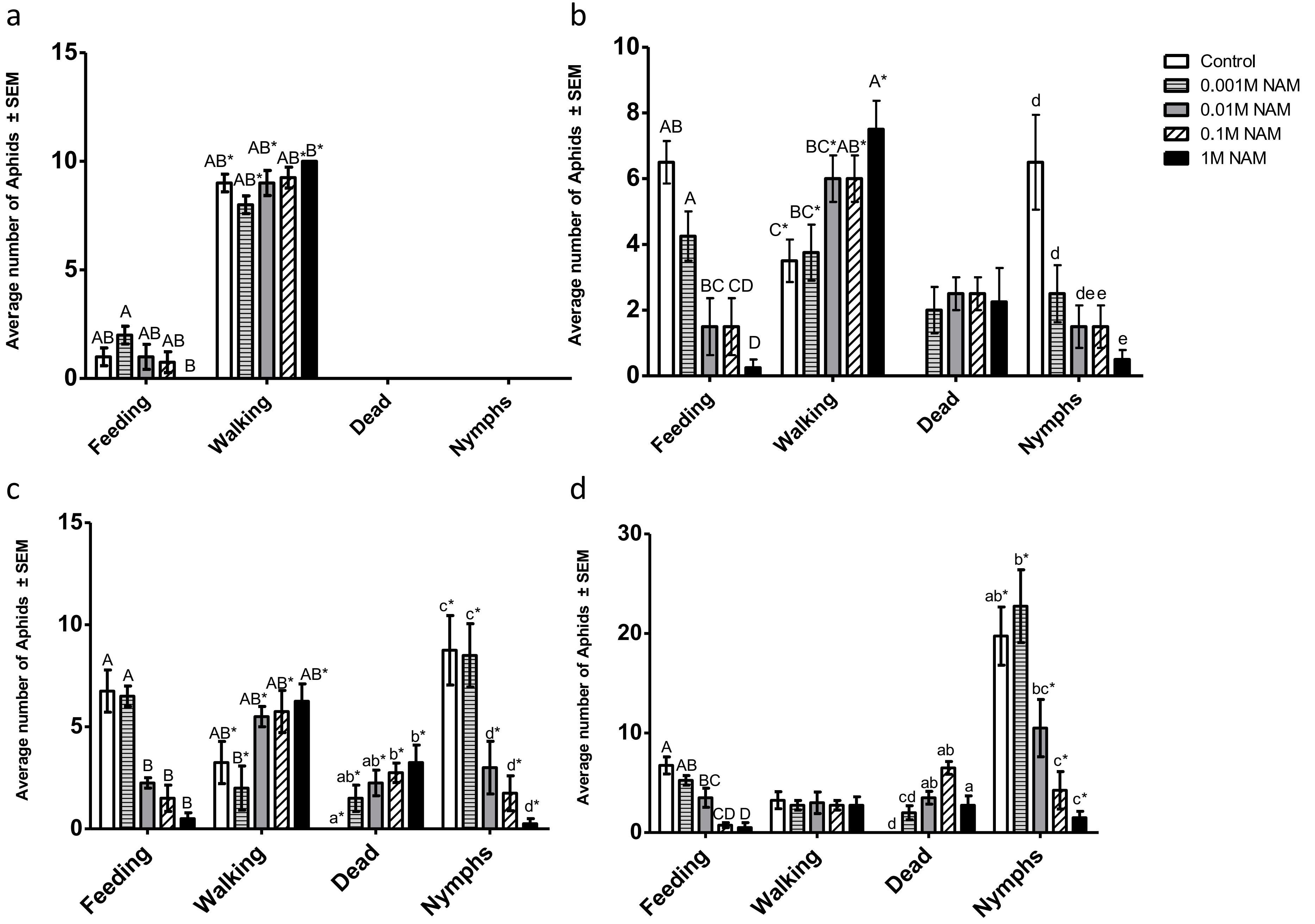

Supplemental Figure S2

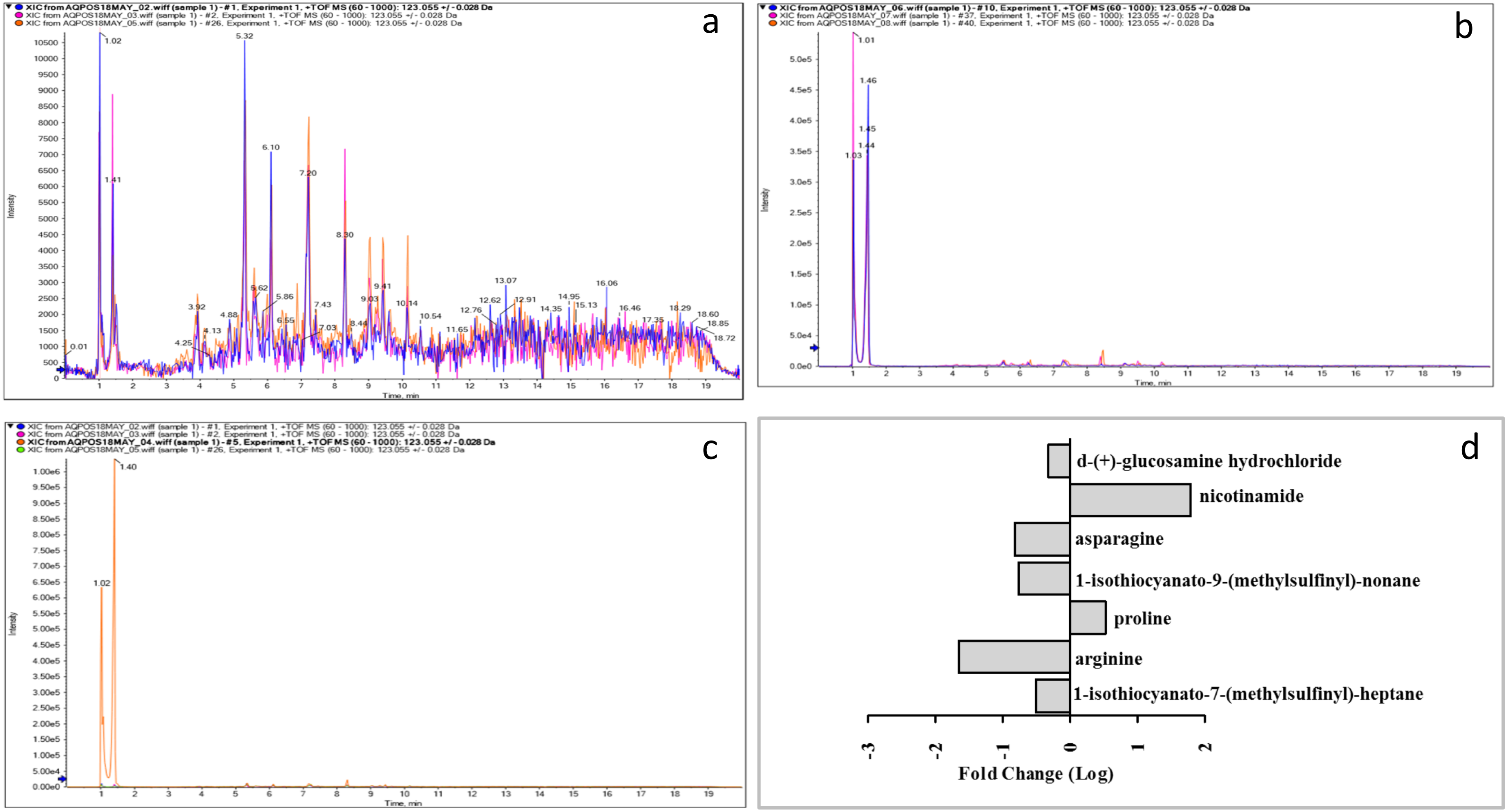

Supplemental Figure S3.

Metabolite name	Average Mz	Average Rt(min)	Fold change in *nic-1-1* plants	P-value
1-isothiocyanato-6-(methylsulfinyl)-hexane	206.0661	9.06	0.284029767	0.213
1-isothiocyanato-7-(methylsulfinyl)-heptane	220.0816	10.41	0.311257035	0
1-isothiocyanato-9-(methylsulfinyl)-nonane	248.1127	12.45	0.170746397	0.008
1h-indole-3-carboxylic acid	162.0548	5.26	0.708806624	0.559
2-acetoxy-4-pentadecylbenzoic acid m+na	413.2657	16.5	1.211669659	0.437
2'-deoxyguanosine 5'-monophosphate	348.0704	1.03	1.029554941	0.571
3-(4-hydroxy-3,5-dimethoxyphenyl)-2-propenoic acid	225.0757	5.16	0.250428478	0.074
3-formylindole	146.0607	1.01	0.796394453	0.541
5-oxo-l-proline	130.0493	0.96	0.535828022	0.296
5'-deoxy-5'-methylthioadenosine	298.0971	3.5	1.400968869	0.22
adenine hydrochloride	136.0614	1.5	0.716546763	0.437
arginine	175.118	0.91	0.022467657	0
asparagine	133.0606	0.94	0.15156671	0.01
aspartate	134.045	0.99	1.117596306	0.654
camalexin	201.0467	9.83	0.239784321	0.046
choline	104.1067	0.92	0.751474597	0.108
d-(+)-glucosamine hydrochloride	180.0866	2.05	0.480368398	0.002
d,l-sulforaphane	178.0355	6.3	0.544422707	0.071
glutamic acid	148.0605	0.96	1.024131138	0.743
glutamine	147.0757	0.95	0.303725798	0.226
indole-3-carbinol	130.0645	5.86	1.165025063	0.539
isoleucine	132.1015	1.5	0.64599385	0.391
kaempferitrin	579.1679	6.57	0.848919416	0.094
kaempferol	287.0538	6.57	0.97332968	0.883
kaempferol-3-o-alpha-l-rhamnoside	433.1135	6.57	1.06199455	0.567
kaempferol-3-o-beta-glucopyranosyl-7-o-alpha-rhamnop	595.1662	5.3	1.059346116	0.73
kaempferol-3-o-rutinoside	617.1425	6	1.258231802	0.336
nandrolone	275.1987	13.41	1.253868606	0.706
ne,ne,ne-trimethyllysine	189.1585	0.9	0.450368469	0.034
nicotinamide	123.0554	1.02 and 1.44	62.03999438	0
norvaline	118.0869	1.01	1.145346752	0.608
pantothenate	220.1177	3.26	0.903384212	0.725
phosphocholine chloride	184.0743	0.98	0.823310105	0.106
proline	116.0707	0.98	3.390987787	0.009
sinapoyl malate	341.0883	4.95	1.546582484	0.275
sn-glycero-3-phosphocholine	258.1098	0.98	0.300962184	0.018
spermidine	146.1633	0.7	1.012148967	0.895
spermine	203.2231	0.7	2.958845129	0.023
threonine	120.0653	0.95	0.6026095	0.193
trans-ortho-coumaric acid	165.0535	1.45	1.025187614	0.941
tryptophan	205.0966	3.19	1.156880084	0.41
tyrosine	182.0809	1.45	0.978850663	0.955
adenosine	268.1045	1.02 and 1.43	0.653432778	0.389
